# From bedside to the bench: patient-specific hiPSC-EC models uncover endothelial dysfunction in genetic cardiomyopathies

**DOI:** 10.3389/fphys.2023.1237101

**Published:** 2023-07-19

**Authors:** Martina Rabino, Elena Sommariva, Serena Zacchigna, Giulio Pompilio

**Affiliations:** ^1^ Unit of Vascular Biology and Regenerative Medicine, Centro Cardiologico Monzino—IRCCS, Milan, Italy; ^2^ Unit of Cardio-Oncology, Centro Cardiologico Monzino—IRCCS, Milan, Italy; ^3^ Cardiovascular Biology Laboratory, International Centre for Genetic Engineering and Biotechnology, Trieste, Italy; ^4^ Department of Medicine, Surgery and Health Sciences, University of Trieste, Trieste, Italy; ^5^ Department of Biomedical, Surgical and Dental Sciences, Università degli Studi di Milano, Milan, Italy

**Keywords:** human induced pluripotent stem cells (hiPSCs), hiPSC-derived endothelial cells (hiPSC-ECs), endothelial dysfunction (ED), genetic cardiomyopathies, disease modelling, personalized medicine

## Abstract

Genetic cardiomyopathies are a group of inherited disorders in which myocardial structure and function are damaged. Many of these pathologies are rare and present with heterogenous phenotypes, thus personalized models are required to completely uncover their pathological mechanisms and develop valuable therapeutic strategies. Both cardiomyocytes and fibroblasts, differentiated from patient-specific human induced pluripotent stem cells, represent the most studied human cardiac cell models in the context of genetic cardiomyopathies. While endothelial dysfunction has been recognized as a possible pathogenetic mechanism, human induced pluripotent stem cell-derived endothelial cells are less studied, despite they constitute a suitable model to specifically dissect the role of the dysfunctional endothelium in the development and progression of these pathologies. In this review, we summarize the main studies in which human induced pluripotent stem cell-derived endothelial cells are used to investigate endothelial dysfunction in genetic-based cardiomyopathies to highlight new potential targets exploitable for therapeutic intervention, and we discuss novel perspectives that encourage research in this direction.

## 1 Introduction

The wide spectrum of genetic cardiomyopathies encompasses different inherited heart muscle disorders characterized by structural and functional abnormalities of the myocardium. According to a scientific statement from the American Heart Association, genetic cardiomyopathies include hypertrophic cardiomyopathy (HCM), dilated cardiomyopathy (DCM), restrictive cardiomyopathy (RCM), arrhythmogenic right ventricular cardiomyopathy (ARVC), and left ventricular noncompaction (LVNC) (Salemi et al., 2021). Briefly, HCM is characterized by left ventricular hypertrophy, DCM involves ventricular dilation, RCM is marked by resistance to ventricular filling, ARVC involves fibrofatty replacement of the right ventricular myocardium, and LVNC is distinguished by the presence of excessive trabeculations within the left ventricle (Muchtar et al., 2017; Wilcox and Hershberger, 2018; Paluszkiewicz et al., 2022). These conditions share certain characteristics, such as highly heterogeneous phenotypes, impaired contractile function, propension to arrhythmic events, and involvement of multiple cellular populations. Genetic cardiomyopathies also include the cardiomyopathy resulting from storage disorders, where metabolic impairment leads to the accumulation of specific substrates, i.e., glycogen in the case of Danon disease and glycosphingolipids in the case of Fabry disease (FD) (Nair et al., 2019). In addition, genetic risk factors influence the probability of an otherwise sporadic cardiomyopathy, like the one resulting from coronary artery disease (CAD), characterized by insufficient blood, oxygen and nutrients supply to the heart muscle due to plaque build-up within the coronary artery wall (Kessler and Schunkert, 2021).

Over the past years, the use of human induced pluripotent stem cells (hiPSCs) has gained increasing recognition in the modelling of these genetically-determined cardiomyopathies. The hiPSC technology indeed allows for the generation of personalized human models, which retain the genetics of the patient of origin, and in which virtually any disease-relevant cell type can be obtained *in vitro* (Takahashi and Yamanaka, 2006; Yu et al., 2022). Furthermore, the possibility of correcting the pathogenic mutation using CRISPR/Cas9-based approaches facilitates the identification of the genotype-phenotype correlation. In this context, hiPSC-derived cardiomyocytes (hiPSC-CMs) and fibroblasts (hiPSC-FBs) are probably the most commonly used cardiac cells. In contrast, hiPSC-derived endothelial cells (hiPSC-ECs) are less explored, despite the critical role played by endothelial dysfunction (ED) in various cardiovascular diseases (Xu et al., 2021).

Mounting evidence suggests that this holds true also for genetic cardiomyopathies. ED pertains to the impairment of key endothelial functions, such as blood vessel formation, maintenance of vascular permeability, thrombosis prevention, regulation of vascular tone, and participation in inflammatory reactions (Michiels, 2003). In HCM patients multiple biomarkers indicative of ED and hemostasis defects have been identified, exhibiting positive correlations with diastolic dysfunction, atrial fibrillation, non‐sustained ventricular tachycardia, left ventricle outflow tract obstruction, bleeding risk, and adverse clinical outcome (Matthia et al., 2022). Although not yet proven, this is consistent with potential impairment of myocardial perfusion and contractility driven by ED. In the context of DCM, impaired vascularization, together with anomalies in both vasculogenesis and angiogenesis, contribute to disease progression and unfavorable prognosis (Roura and Bayes-Genis, 2009). Furthermore, these patients exhibit diminished levels of endothelial progenitor cell-colony forming units, along with impaired endothelial-dependent vasodilation during flow-mediated dilation (FMD) test (Premer et al., 2015). In a case report of a patient with RCM complicated by myocarditis, thickening of the atrial wall and pulmonary vein orifices was observed, associated with the presence of an inflammatory infiltrate rich in giant cells (Law et al., 2004). This finding may suggest a link between ED, in particular enhanced permeability to inflammatory infiltrates, and RCM progression. In addition to the common forms of genetic cardiomyopathies mentioned above, ED has been implied in the pathogenesis of other inherited diseases, including Danon disease, hereditary amyloidosis and long QT syndrome (LQTS) (Saftig et al., 2001; Al-Zaiti et al., 2018; Nguyen et al., 2018; Hummel et al., 2020; Koike and Katsuno, 2020). Furthermore, ED has also been recognized as a contributing factor in other genetically determined diseases affecting the cardiovascular system, including FD and genetic forms of CAD (Shen et al., 2008; Rombach et al., 2012; Matsuzawa and Lerman, 2014). Given these evidences, it becomes crucial to delve deeper into the pathogenic mechanisms underlying human ED in genetic cardiomyopathies. In this context, hiPSC-ECs represent a precious tool to uncover novel potential therapeutic targets.

**FIGURE 1:**
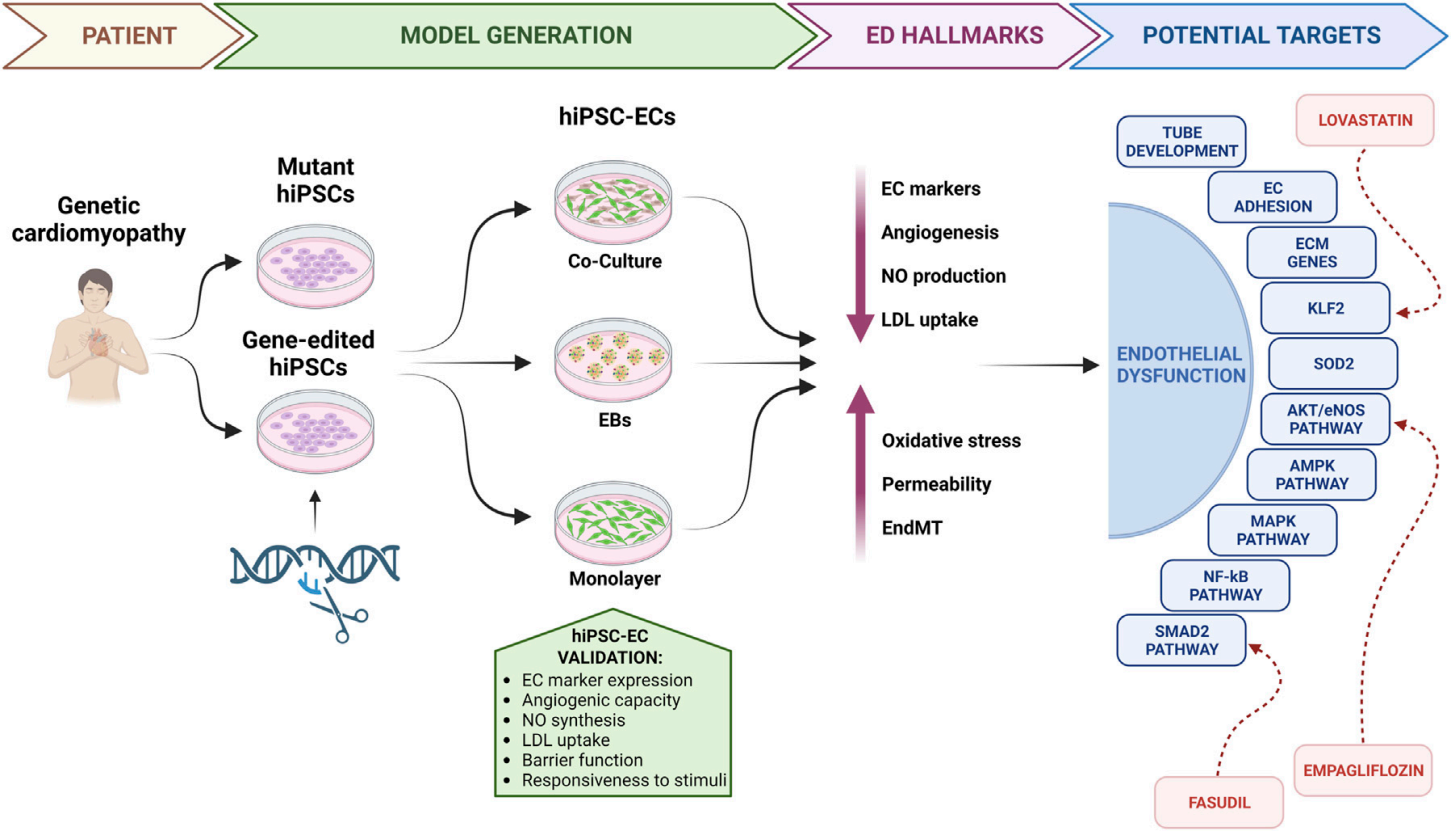
Endothelial dysfunction in genetic cardiomyopathies: from bedside to the bench. hiPSCs, human induced pluripotent stem cells; hiPSC-ECs, human induced pluripotent stem cell-derived endothelial cells; EBs, embryoid bodies; ED, endothelial dysfunction; EC, endothelial cell; NO, nitric oxide; LDL, low density lipoprotein; EndMT, endothelial-to-mesenchymal transition; ECM, extracellular matrix.

To stimulate research in this direction, in this review we will summarize the most commonly used techniques to generate and validate hiPSC-ECs, examine their suitability in modelling ED in genetic cardiomyopathies, highlighting new potential therapeutic targets, and discuss the implications and potential advancements in this field (Figure 1).

## 2 Generation and validation of hiPSC-ECs

The generation of ECs from hiPSCs has gained significant attention in recent years due to its potential applications in disease modelling and regenerative medicine. A range of methodologies have been developed, mainly taking advantage of three approaches: co-culture with stromal cells, embryoid body (EB) formation and monolayer differentiation. Comprehensive reviews on these methods have been published recently (Lin et al., 2017; Kennedy et al., 2021; Trillhaase et al., 2021).

Briefly, co-culture systems involve the culture of hiPSCs with other cell types exerting a supportive function, by providing paracrine signals that facilitate the differentiation process. As an example, the first hiPSC-ECs were generated in 2009 on a feeder layer composed of murine bone marrow-derived stromal cells (OP9) (Choi et al., 2009; Taura et al., 2009). While this achievement was remarkable, the approach proved to be relatively inefficient, labor-intensive, and unsuitable for clinical purposes due to the presence of murine contaminants. To address some of these limitations, the EB formation-based differentiation method was developed, which mimics early embryonic development. In this set-up, hiPSCs are seeded into ultra-low attachment culture plates to favor the spontaneous formation of cellular aggregates, that are then allowed to adhere and undergo endothelial specification, under the influence of specific growth factors. Li et al. and Rufaihah et al. pioneered this feeder-free approach, using either a combination of vascular endothelial growth factor (VEGF) and basic fibroblast growth factor (bFGF), or solely VEGF, to specify endothelial fate (Li et al., 2011; Rufaihah et al., 2011). Also in this case, the efficiency remained low and the generated hiPSC-ECs, despite expressing some specific EC markers, were highly heterogeneous. Currently, the most commonly employed approach to achieve EC differentiation from hiPSCs relies on monolayer cultures with addition of small molecules. This strategy entails culturing hiPSCs as a monolayer on matrix-coated culture plates and administering targeted small molecules to first induce mesodermal differentiation and subsequent drive cells towards EC fate. To obtain a pure EC population, cell sorting techniques, either fluorescence- or magnetic-activated, are commonly employed as a final step. Using this approach, Park et al. showcased the successful attainment of EC differentiation in an animal serum- and feeder-free system, by synergistically regulating two signaling pathways, namely, MEK/ERK and BMP4 (Park et al., 2010). Building upon these findings, a number of small molecule combination have emerged, resulting in increasingly efficient, robust, cost-effective, and time-efficient protocols (Kennedy et al., 2021). In addition, precise modulation of small molecules and factors allows for the generation of distinct subtypes of hiPSC-ECs, such as arterial, venous, or lymphatic ECs, thus enabling to generate homogeneous populations that closely resemble their *in vivo* counterparts (Lee et al., 2015; Rosa et al., 2019). Further EC maturation and functionality can be achieved by the integration of diverse cell types, such as hiPSC-ECs and hiPSC-CMs (Giacomelli et al., 2017a) within 3D models, i.e., microtissues (MTs) and organoids (Giacomelli et al., 2017b; Lewis-Israeli et al., 2021; Kahn-Krell et al., 2022; Voges et al., 2023), as discussed later. Regardless of the methodology used for their generation, hiPSC-EC validation is crucial to ensure their authenticity and functional competence. Several key validation criteria are commonly employed to confirm the endothelial identity of hiPSC-derived cells. First, immunophenotyping using either flow cytometry or immunofluorescence is needed to assess the expression of specific endothelial markers, such as CD31, von Willebrand factor (vWF), vascular endothelial cadherin (VE-cadherin), and endothelial nitric oxide synthase (eNOS) (Garlanda and Dejana, 1997; Li et al., 2008). Then, functional assays have to be conducted to evaluate the angiogenic potential of hiPSC-ECs, such as their ability to form tube-like structures on Matrigel or hydrogel, and/or to support vasculogenesis *in vivo* in xenograft models (DeCicco-Skinner et al., 2014; Orlova et al., 2014; Belair et al., 2016). Functional validation should also include assays for nitric oxide (NO) synthesis, low-density lipoprotein (LDL) incorporation, and for endothelial barrier function, such as transendothelial electrical resistance (TEER) or permeability assays using fluorescent tracers (Ritter et al., 2018; Goshi et al., 2019; Fengler et al., 2022). Moreover, hiPSC-ECs should respond to relevant stimuli, such as VEGF, by activating downstream signalling pathways involved in angiogenesis (Zachary, 2003). Collectively, extensive validation of hiPSC-ECs is recommended to ensure their functionality and suitability for various applications in disease modelling, drug discovery, and regenerative medicine.

## 3 Modelling genetic cardiomyopathies with hiPSC-ECs

Thus far, a limited number of studies used hiPSC-ECs derived from patients with genetic cardiomyopathies to examine the contribution of ED to disease onset and progression. Most of these studies, listed in Table 1, are presented and discussed in the next paragraphs.

**TABLE 1:** hiPSC-EC-based models of genetic cardiomyopathies.

Disease	Gene/SNP	Model	Phenotype	Mechanism/Pathway	Treatment	References
DCM	*LMNA*	- *LMNA* hiPSC-ECs - Isogenic *LMNA* hiPSC-ECs - *LMNA*-corrected hiPSC-ECs	- Reduced EC marker expression - Impaired angiogenesis - Reduced NO production - Reduced Ac-LDL accumulation	- KLF2 as a potential transcription factor responsible for EC dysfunction	- Lovastatin rescued EC dysfunction	Sayed et al. (2020)
FD	*GLA*	- FD hiPSCs-ECs - WT hiPSC-ECs	- Intracellular Gb3 accumulation - ROS production	- *SOD2* downregulation - AMPK activity enhanced	N/A	Tseng et al. (2017)
FD	*GLA*	- FD hiPSC-ECs - WT hiPSC-ECs - *TSP-1*- KO hiPSC-ECs - Isogenic control hiPSC-ECs	- Intracellular Gb3 accumulation - Impaired angiogenesis - Metabolic dysfunction - Oxidative stress	- TSP-1 upregulation - SMAD2 signalling enhanced	N/A	Do et al. (2020)
FD	*GLA*	- Treated FD hiPSC-ECs - Untreated FD hiPSC-ECs	- Defective tube formation - Increased EndMT - Impaired metabolic processes	- SMAD2 signalling	- Fasudil improved vascular endothelial function, LVH, anhidrosis, heat insensitivity, and renal fibrosis	Choi et al. (2023)
FD	*GLA*	- FD hiPSC-ECs - Isogenic control hiPSC-ECs	- Intracellular Gb3 accumulation - Autophagic flux impairment - ROS production	- Inflammation-related NF-κB and MAPK pathways - Tube development pathways	N/A	Song et al. (2021)
Genetic CAD	SNPs in the 9p21.3 CAD risk locus	- Risk haplotype hiPSC-ECs - Non-risk haplotype hiPSC-ECs - Isogenic KO hiPSC-ECs	- Greater vessel permeability - ROS signalling	- Differential regulation of EC adhesion and ECM genes	N/A	Teng et al. (2021)
Genetic CAD	*ALDH2 rs671*	- *ALDH2 rs671* hiPSC-ECs - *ALDH2 rs671* corrected hiPSC-ECs - WT hiPSC-ECs	- Oxidative stress - Inflammation - Reduced NO production - Impaired angiogenesis	- Inhibition of AKT/eNOS pathway	- Empagliflozin mitigated *ALDH2 rs671*-associated EC dysfunction	Guo et al. (2023)

DCM, dilated cardiomyopathy; hiPSC-ECs, human induced pluripotent stem cell-derived endothelial cells; EC, endothelial cell; NO, nitric oxide; Ac-LDL, acetylated-low density lipoprotein; FD, Fabry disease; WT, wild type; ROS, reactive oxygen species; N/A, not applicable; EndMT, endothelial-to-mesenchymal transition; LVH, left ventricular hypertrophy; CAD, coronary artery disease; SNP, single nucleotide polymorfism; KO, knock out; ECM, extracellular matrix.

In the case of DCM caused by mutation in the *LMNA* gene, Sayed et al. demonstrated several hallmarks of ED in hiPSC-ECs from patients with two different *LMNA* variants. Regardless of the mutation, *LMNA* hiPSC-ECs displayed impaired EC phenotype and function, characterized by lower expression of EC markers and reduced ability to form capillary network, produce NO, and accumulate acetylated LDL (Sayed et al., 2020). This phenotype was confirmed in ECs differentiated from genome-edited hiPSCs, thus providing a causal link between *LMNA* mutations and ED. Most importantly, the presence of these defects in the diseased hiPSC-ECs correlated with the severity of clinical ED documented in the enrolled *LMNA* patients, in which hyperaemia studies showed reduced EC function. In this work, the authors not only recapitulated the pathological phenotype, but also identified Krüppel-like factor 2 (KLF2) as a potential transcription factor responsible for ED. Finally, they showed that treatment of *LMNA* hiPSC-ECs with lovastatin, a KLF2 agonists, rescued the phenotype, mirroring the benefit observed in patients treated with the same drug. Overall, these data confirm that hiPSC-ECs can successfully model ED, and allow to perform mechanistic and personalized medicine studies (Sayed et al., 2020).

Among genetically determined diseases affecting the cardiovascular system, FD is a X-linked lysosomal storage disorder caused by pathogenic variants in the *GLA* gene that results in reduced α-galactosidase A (α-Gal A) enzyme activity and consequent accumulation of globotriasylceramide (Gb3) in a wide range of cells, including ECs (Ortiz et al., 2018). Implications of the cardiovascular compartment typically include left ventricular hypertrophy, myocardial fibrosis, heart failure and arrhythmias, all signs of advanced cardiomyopathy (Pieroni et al., 2021). In this context, Tseng et al. pioneered the development of a hiPSC-derived vascular endothelial model to investigate ED associated with FD (Tseng et al., 2017). They successfully differentiated vascular ECs from hiPSCs carrying the *GLA IVS4+919G>A* mutation and showed that FD hiPSC-ECs recapitulate a major disease hallmark by accumulating Gb3 in the lysosomal compartment, as witnessed in cardiac biopsies obtained from the affected subjects. Taking advantage of this patient-specific endothelial model, the researchers found that the amplified reactive oxygen species (ROS) production detected in FD hiPSC-ECs, driven by Gb3 accumulation, was accompanied by a decrease in SOD2 and an increase in AMPK signalling, respectively. As AMPK activation was shown to protect from oxidative stress in human endothelium (Mackenzie et al., 2013), further investigation is warranted to explore the interaction between AMPK, mitochondrial SOD2 and imbalanced redox status in FD. In a different study, Do et al., proposed a distinct mechanism wherein enhanced expression of thrombospondin-1 (TSP-1), coupled with the hyperactivation of SMAD signaling, contributes to impaired angiogenesis and altered transcription of oxidative stress-related genes detected in FD hiPSC-ECs (Do et al., 2020). Few years later, the same group demonstrated that fasudil alleviates ED in both FD hiPSC-ECs and mice, by downregulating TSP-1 and SMAD signalling (Choi et al., 2023). If confirmed, these data could pave the way to an additional treatment for FD patients, who at present can only benefit from enzyme replacement therapy, which is effective but fraught by major drawbacks, including partial tissue permeation, generation of anti-drug antibodies, and high cost (Azevedo et al., 2020). So far, Song et al. demonstrated a causal relation between the *GLA IVS4+919G>A* mutation and the occurrence of ED by establishing hiPSC lines in which the mutation was corrected using CRISPR/Cas9 technology (Song et al., 2021). The pathological phenotypes detected in FD hiPSC-ECs, i.e., intracellular Gb3 accumulation, autophagic flux impairment and ROS production were rescued in the corrected isogenic lines. Furthermore, the authors took advantage of the generated tools to gain insight on novel mechanisms. In particular, transcriptomic analyses showed that the *GLA IVS4+919G>A* mutation mainly altered the expression of genes belonging to inflammatory and angiogenic pathways.

In a broader view of genetic influence on cardiomyopathies, CAD and the subsequent ischemic cardiomyopathy is worth a mention. Several large-scale genomic studies, previously reviewed (Kessler and Schunkert, 2021), identified a number of genomic loci associated with this pathology, thus confirming the role of a genetic component, in addition to environmental and lifestyle-related factors, in the development of CAD. In this context, hiPSC-ECs were implied to investigate the effects of the 9p21.3 CAD risk locus at the cellular level. Teng et al. reported that hiPSC-ECs carrying the risk haplotype in homozygosity (*R/R* WT) exhibit enhanced permeability and ROS signalling, compared to knockout isogenic lines edited by TALENs (*R/R* KO) (Teng et al., 2021). One of the strengths of this work is the use of two different hiPSC-EC-based models with increasing level of complexity. Indeed, by generating a 3D microfluidic device containing a microvessel within a collagen scaffold, the authors were able to investigate the effect of laminar shear stress on permeability and transcription. Being in a more physiologically relevant environment, they demonstrated a higher permeability of *R/R* WT vessels in response to shear stress, as well as a greater tendency of hiPSC-ECs subjected to shearing forces to cluster based on risk haplotypes, upon transcriptome analysis. This led to a significant differential expression of EC adhesion and extracellular matrix genes, compared to static conditions. Another study applied a similar approach to dissect the role of a single nucleotide polymorphism (SNP) in a non-coding locus on endothelial function and its relation to CAD. The SNP under investigation was *ALDH2 rs671*, which was known to be associated with a high risk of CAD, but without a clear mechanism (Li et al., 2018). Carriers of the variant showed compromised blood vessel function and *ALDH2 rs671* hiPSC-ECs reflected ED *in vitro*, as documented by a cascade of increased oxidative stress, inflammation, reduced NO production, and compromised tube formation capacity (Guo et al., 2023). To dive deeper into the underlying mechanisms, the authors investigated the transcriptional profile of *ALDH2 rs671* hiPSC-ECs and their isogenic counterpart, and found that this variant resulted in inhibition of the AKT/eNOS pathway. Interestingly, the sodium-glucose cotransporter 2 inhibitor (SGLT2i) empagliflozin was able to restore the phenotype, not only *in vitro* but also *in vivo* in *ALDH2 rs671* mice. Therefore, using hiPSC-ECs, Guo et al. were able to show that empagliflozin exerted its beneficial effect on ED leveraging of a “non-canonical” mechanism of action. Consistent with the lack of SGLT expression by hiPSC-ECs, the empagliflozin positive outcome is likely mediated by inhibition of Na^+^/H^+^ exchanger isoform-1 (NHE-1) activity, followed by restoration of both AKT and eNOS signaling.

## 4 Discussion

In this manuscript, we have focused on the role of ED in genetic cardiomyopathies, discussing all the studies to our knowledge that have exploited hiPSC-ECs to model ED in these pathologies.

From a clinical perspective, significant associations have been found between ED and pathological phenotypes, disease progression, and unfavourable clinical outcome in most of the major cardiomyopathies, namely, HCM, DCM and RCM (Law et al., 2004; Roura and Bayes-Genis, 2009; Matthia et al., 2022). ED has also been described in the cardiomyopathy resulting from genetically influenced CAD, as well as in genetically determined storage diseases, i.e., Danon disease and FD (Saftig et al., 2001; Shen et al., 2008; Rombach et al., 2012; Matsuzawa and Lerman, 2014; Nguyen et al., 2018; Hummel et al., 2020). Nevertheless, other prominent cardiomyopathies, including ARVC and LVNC, remain relatively understudied in this context, creating a gap in our understanding. Conducting investigations for these cardiomyopathies would be intriguing to determine if ED also plays a role, as in the others.

From a preclinical standpoint, genetic cardiomyopathies have been the subject of intensive investigation in diverse animal models (Gerull and Brodehl, 2020; Solomon et al., 2021; Chintanaphol et al., 2022; Kaur et al., 2023); however, the role of ED has been largely overlooked. Nonetheless, it is crucial to acknowledge that these models may provide partial answers, as they may not fully recapitulate the human phenotype. A striking example is the aggressive form of ARVD caused by the S358L mutation in the *TMEM43* gene. While Luma S358L KI mice exhibited normal cardiac function, hiPSC-CMs carrying the same mutation show contraction abnormalities, including increased contraction duration, time to peak, and relaxation time, along with decreased contraction amplitude (Stroud et al., 2018; Padron-Barthe et al., 2019). The hiPSC technology offers a valuable alternative in this respect. Being derived from either healthy or diseased individuals, hiPSC-ECs provide data that are highly relevant to human physiology and pathology, thus circumventing interspecies pathophysiological variations. Furthermore, patient-specific hiPSC-ECs retain the genetic background and characteristics of the individual they were derived from, thereby enabling personalized medicine approaches. The possibility of edit their genome using CRISPR/Cas9 approach, which is by far less efficient in primary ECs, allows to assess the specific effect of mutations, establishing a causal link with the pathological phenotype. In addition, unlike primary ECs, which necessitate invasive procedures for isolation and possess limited proliferative capacity, hiPSC-ECs can be obtained non-invasively and extensively expanded, thus allowing more rigorous and reproducible results. They also provide relevant advantages over human umbilical vein endothelial cells (HUVECs), which are commonly used to study ED in humans. Indeed, the targeted induction of cardiac mesoderm prior to specification into EC fate enables the generation of hiPSC-ECs which preserve organotypic specificity and share multiple features with primary cardiac ECs.

Despite these advantages, the utilization of hiPSC-ECs for investigating ED in genetic cardiomyopathies has been limited so far. To our knowledge, among the major genetic cardiomyopathies, only LMNA-associated DCM has been studied in this respect (Sayed et al., 2020). Other studies investigating ED with hiPSC-ECs refer to the field of genetically determined diseases also affecting the cardiovascular system, such as FD and genetic predisposition to CAD (Tseng et al., 2017; Song et al., 2021; Teng et al., 2021; Guo et al., 2023). Although these studies are few in number, they have yielded valuable scientific insights. First of all, they have allowed for the specific isolation and characterization of pathological phenotypes in hiPSC-ECs, revealing common features of ED in different genetic cardiomyopathies, including impaired angiogenesis, reduced NO production, increased ROS production and altered vessel permeability. Furthermore, these investigations have led to the identification of potential molecular factors and pathways involved in ED in the specific disease context, such as KLF2, SOD2, EC adhesion and ECM genes, as well as AMPK, NF-κB, MAPK, AKT/eNOS, SMAD and tube development pathways. Lastly, some targeted therapeutic approaches aimed at rescuing EC dysfunctions have emerged, i.e., lovastatin in the case of LMNA-DCM, fasudil for FD and empagliflozin in *ALDH2 rs671*-associated CAD. Given the available data and the observation that the genes responsible for the mentioned cardiomyopathies are also expressed in ECs, it becomes clear that there is a direct, cell-autonomous connection between ECs and these pathologies. Whether the intrinsic defect in ECs acts as the trigger for dysfunction in other cell types, primarily CMs, or it is rather a consequence, remains an outstanding question. The two hypotheses are not mutually exclusive, as ECs and CMs engage in bidirectional communication through paracrine signaling, cell-cell interactions, and extracellular matrix remodeling (Colliva et al., 2020). Regardless of the triggering factor, it is plausible that dysfunctional ECs disrupt the physiological crosstalk between resident cardiac cells, thus contributing to the pathology. Clearly this cannot be verified in a model relying solely on hiPSC-ECs, and the adoption of a multicellular approach is both essential and underutilized. These limitations constitute a noteworthy aspect of the studies discussed in this review. Furthermore, the use of hiPSC-ECs for disease modelling presents additional constraints, including their immaturity, the potential retention of original tissue-specific memory, and the heterogeneity of the resulting cell population encompassing arterial, venous, and/or lymphatic ECs. Reproducibility and variability are also pertinent considerations, as the differentiation of hiPSCs into ECs involves a multifaceted and variable process, leading to discrepancies in the characteristics of generated hiPSC-EC populations.

Several factors may contribute to the underutilization of hiPSC-ECs in the context of genetic cardiomyopathies. Firstly, the focus has predominantly been on studying hiPSC-CMs, in which the genetic defect was considered for its direct impact on contractile function. Secondly, the establishment of robust protocols for hiPSC differentiation into functional ECs has lagged behind hiPSC-CM differentiation methods. Lastly, the complexity of ED and its interplay with other cell types in the heart is difficult to reproduce *in vitro*. Different attempts have been made via hiPSC-ECs in MTs, engineered heart tissues (EHTs) or organoids, although the focus of the studies was not specifically ED. Rather, the emphasis has been on obtaining complex physiologic models to recapitulate the cardiac features in a dish or disease models, to get insights on the pathogenesis. These models include direct and paracrine crosstalk between ECs and CMs (Giacomelli et al., 2017b), either alone or in combination with other cardiac cell types (Mills et al., 2017; Kahn-Krell et al., 2022). This is known to increase CM maturation, possibly due to cardiac endothelium cardiotropic function (Segers et al., 2018), and to induce vascularization. Only few studies, in which hiPSC-EC were included in a MT or EHT, have modelled cardiomyopathies, such as ACM (Giacomelli et al., 2020), LQTS (Giacomelli et al., 2021), and Duchenne muscular dystrophy-associated cardiomyopathy (Marini et al., 2022), with no specific reference to ED and its effect on the tissue *in vitro*. In addition, most cardiac organoids, obtained by differentiation and self-organization of hiPSCs show a proportion of ECs (Mills et al., 2017; Lewis-Israeli et al., 2021). Also in this case, ECs have been shown improve cardiac organoid structure and function (Voges et al., 2023), even if no specific data on ED are available. Focusing efforts in this area will be crucial to fill this knowledge gap. By incorporating patient-specific mutations utilizing genome editing techniques, researchers can investigate the specific effects of a mutation on EC function and their contribution to the pathogenesis of cardiomyopathies.

In conclusion, while the role of ED in genetic cardiomyopathies is increasingly recognized, the use of hiPSC-ECs as a model system to study this aspect remains limited. Expanding investigation of hiPSC-ECs in genetic cardiomyopathies can provide valuable insights into the pathogenesis of these disorders and identify new potential therapeutic targets. Further research in this direction is warranted to harness the full potential of hiPSC-ECs and advance our understanding of ED in the context of genetic cardiomyopathies.
